# Establishing the original order of the poems in Harward’s Almanac using paleography, codicology, X-ray fluorescence spectroscopy, and statistical analysis

**DOI:** 10.1186/s40494-023-01107-y

**Published:** 2023-12-15

**Authors:** Veronica Biolcati, James Woolley, Élodie Lévêque, Andrea Rossi, Anna Grace Hoffmann, Andrea Visentin, Pádraig Ó Macháin, Daniela Iacopino

**Affiliations:** 1 Tyndall National Institute, University College Cork, Lee Maltings Complex, Dyke Parade, Cork, T12R5CP Ireland; 2 Department of English, Lafayette College, Easton, PA 18042 USA; 3 Centre de Recherche Histoire Culturelle et Sociale de l’Art (HiCSA), Université Paris 1 Panthéon-Sorbonne, 2 rue Vivienne, 74002 Paris, France; 4 School of Computer Science & IT, Centre for Research Training in Artificial Intelligence, University College Cork, Western Gateway Building, Western Road, Cork, T12 XF62 Ireland; 5 Modern Irish Department, University College Cork, College Road, Cork, T12K8AF Ireland

**Keywords:** Paleography, Codicology, Manuscript studies, Inks, Paper, X-ray fluorescence, Conservation, Statistical analysis, Clustering

## Abstract

This work presents the results of a transdisciplinary analysis performed on Harward’s Almanac (Dublin, 1666), an extremely rare volume currently housed in the National Library of Ireland. The uniqueness and historical value of the Almanac is related to the presence of nineteen handwritten poems, entered by an anonymous scribe. These record textually important English clandestine satire circulating anonymously in Dublin in the late seventeenth and early eighteenth century. Following a comprehensive historical assessment, it appeared evident that the current order of leaves was incorrect. To reconstruct the correct order of the leaves, and hence the likely sequence in which the manuscript poems were inscribed, this study employed a codicological/paleographic analysis complemented by analytical (X-ray fluorescence, XRF) and statistical (Self Organizing Map, SOM) investigation. Specifically, point XRF analysis was carried out for each handwritten page of the Almanac, allowing identification of ink elemental compositions (iron-based ink) and successfully supporting the validity of historical hypotheses on the poems’ order of inscription. The statistical organization of XRF data by SOMs allowed easy bi-dimensional visualization of the data set (54 points) and identification of ink similarities, once more validating the historical assessment.

**Supplementary Information:**

The online version contains supplementary material available at 10.1186/s40494-023-01107-y.

## Introduction

In 2020, the National Library of Ireland (NLI) acquired a volume of extreme rarity, the only known copy of a 1666 Dublin almanac compiled by Michael Harward and for convenience referred to as Harward’s Almanac [1]. Almanacs of the period were commonly sold with blank interleaves and additional blank leaves—at the end of the volume—meant for the purchaser’s notes and memoranda. In Harward’s Almanac, most of these blank leaves, along with the blank regions of some of the printed leaves, remained blank during 1666, but they were later used by an unknown scribe to transcribe nineteen manuscript poems. Although ongoing research highlighted that these poems were composed between 1674 and 1711, it is less clear when they were copied into the Almanac [2]. When the Almanac was first reported in print in 1848, the handwritten poems were erroneously attributed to the Irish satirist Jonathan Swift (1667–1745). Although this attribution was later dismissed [3, 4], the verse retains considerable interest. In fact, the manuscript writing preserves textually important English clandestine satire circulating anonymously in Dublin in the late seventeenth and early eighteenth century. Some of the most important poems are on Dublin topics, and three of them are not found in any other early manuscript or printed source (“The Gentlemen at Larges Litany,” “To the Tune of Chivie Chace,” and “The Picture of a Dublin Beau”). Much of the London verse centers on James II’s Catholicism and the succession crisis caused by the 1688 birth of his son James Francis Edward, later known as the Pretender. These pieces are of interest in their own right and demonstrate the collection and possibly the circulation of London satiric verse in Dublin. No other Dublin manuscript poetical miscellany from the late seventeenth century has come to light, and this stands as the only example [5]. The Almanac was privately acquired sometime in the early nineteenth century and remained in the same Dublin family until it was sold at auction in 2020. As it was received at NLI, Harward’s Almanac was composed of 75 leaves (144 × 86 mm) in a nineteenth-century leather binding. Several leaves were detached from the structure and obviously not in the correct order. Early handwritten page numberings also appeared incorrect.

An extensive historical and codicological analysis was performed which established the original order of the Almanac leaves and thereby supported a plausible idea of the sequence of poem entries. During the 2021–22 conservation work performed by the NLI, the nineteenth-century binding was reused and, with minor exceptions, the leaves were restored in the possible order in which they were placed during the previous (nineteenth-century) rebinding.

In parallel, X-ray fluorescence (XRF) analysis was performed on the writing inks to identify their inorganic elemental compositions. XRF analysis is a non-invasive method particularly useful in the examination of historical manuscripts and inks [6–8]. Among the diverse range of analytical methods used in heritage and conservation science, XRF stands out as a particularly valuable tool for the elemental characterization of materials. The technique offers numerous advantages, including its compatibility with portable instrumentation, which greatly facilitates in situ analysis. XRF requires minimal preparation of the material to be analyzed, making it an efficient and time-saving method. XRF permits qualitative analysis, providing valuable insights into the materials used in historical books and manuscripts. Furthermore, a semi-quantitative analysis of XRF data has been proven effective in uncovering ink patterns, as evidenced by previous studies that have successfully identified various writer contributions and manuscript corrections [9, 10]. The fundamental parameter (FP) is the preferred method for identifying the elemental concentration of heterogeneous samples in complex matrices, such as ink applications [11, 12]. The results obtained using FP quantification can be further analyzed using statistical methods, which can be highly valuable in identifying trends and correlations among samples. One such method is Self Organizing Map (SOM) clustering [13]. SOM mapping is a powerful technique for visualizing high-dimensional data and for clustering (grouping based on features’ similarity) samples in datasets. Without requiring prior knowledge or labeled samples, SOM leverages unsupervised learning to discover the underlying structure and relationships in the data, providing insights and facilitating further analysis. SOMs are based on the concept of neural networks and are inspired by the organization of neurons in the brain. SOMs consist of an array of artificial neurons arranged in a grid-like topology, where each neuron represents the representative point of a group. A successful example of SOM use in heritage science is the work of Lee et al., who classified a large number of paintings into different genres based on image features, including colors and composition characteristics [14, 15].

In this paper we report the use of a transdisciplinary approach based on complementary historical, analytical, and statistical analyses to identify the possible order in which the poems were inscribed in Harward’s Almanac. Following historical and codicological research, a systematic analytical investigation of the Almanac’s handwritten poems was performed using point XRF and microscopy imaging. The ink and the paper support of every manuscript page were elementally characterized. This enabled the identification of calcium-based compounds and alum used for the preparation of the rag paper and iron-based inks for writing the poems. XRF semi-quantitative analysis of ink composition was performed and the resulting data were further analyzed by SOM, in order to identify relevant similarities associated with the stints of transcription. Following this novel approach, it was possible to confirm the historical hypotheses formulated during the Almanac’s conservation work related to the correct leaf sequence and order of poem transcription. This study highlights the usefulness, in the conservation and preservation of heritage manuscripts, of a transdisciplinary approach, which in this specific case sits at the intersection of analytic bibliography, Irish studies, manuscript conservation, heritage science, and computer science.

## Experimental methods

### X-ray fluorescence spectroscopy (XRF)

XRF was performed with a portable, energy-dispersive XRF ELIO (XGLab, srl). The spectrometer was equipped with a Peltier-cooled Silicon Drift Detector with a resolution of 135 eV at the manganese (Mn) Kα line (5.9 keV). The excitation source was a low-power (4 W, 50 kV), transmission X-ray tube with a Rh anode. Measurements were performed with no filter, under air atmosphere in a range 1–25 kV. The tube current was kept at 160 µA. A collimator, allowing a ~ 1 mm diameter focused spot size on the surface of the pages, was used to acquire XRF spectra. The distance from the folio was ~ 1 cm and the acquisition time was 120 s. A semi-quantitative approach to the XRF data was performed using the open-source software PyMca. The so-applied fundamental parameter method (FPM) allowed estimating elements’ concentration based on observed peak intensities. The elemental concentrations of the blank paper areas were subtracted from the ones in inked areas. The areas for XRF analysis were selected following a visual assessment in transmitted light to identify regions without any ink application on the other side of the page. The same process was applied to identify clean paper areas.

### Semi-quantitative XRF analysis using fundamental parameter (FP)

In order to discriminate writing inks and identify manuscript passages containing inks of similar elemental composition, a standardless semi-quantitative approach was applied to the XRF spectra. The conversion of peak intensities to elemental concentration was obtained by applying a FP approach to spectral analysis using the open-source software PyMca. The XRF spectrometer was fully characterized elsewhere, and a configuration file for data processing via PyMca was provided to the authors [16]. The fit parameters were tailored based on the experimental parameters. After having ensured proper fitting for every spectrum, the “batch fitting” and the “RGB correlator” tools were used in order to batch fit the spectra and to find relative ratios of elements (for more details see Additional file 1). For better comparison, all XRF data were normalized with respect to Fe K series peaks.

### Self Organizing Map (SOM)-based clustering

In the analysis, all the characteristic elements in inked areas were subjected to initial analysis using SOM clustering. However, for the final clustering, only the relative concentrations (in relation to Fe) of trace elements Cu and Zn were considered, as these were considered significant to obtain ink distinction. The analysis was conducted using Google Colab with Python 3.10. The Pandas package was utilized for data handling and pre-processing, while Scikit-learn was employed for machine learning and data reduction implementations. The SOM clustering algorithm was implemented using the MiniSom package, and visualizations were generated using Matplotlib. To ensure reproducibility, the code used in the analysis has been made freely available [17].

## Results and discussion

The initial phase of this analytical work involved conducting historical research on the printed Almanac. This was followed by a meticulous codicological observation during the 2021–22 conservation phase at the NLI. The disbound state of the volume during the conservation treatments provided access to typically concealed areas, including the spine sewing, the folding of the sheets, and the sewing holes. The combination of these two perspectives provided valuable information and laid the foundation for the complementary analytical and statistical investigation.

### What we know about the book from historical and codicological analyses

**Fig. 1:**
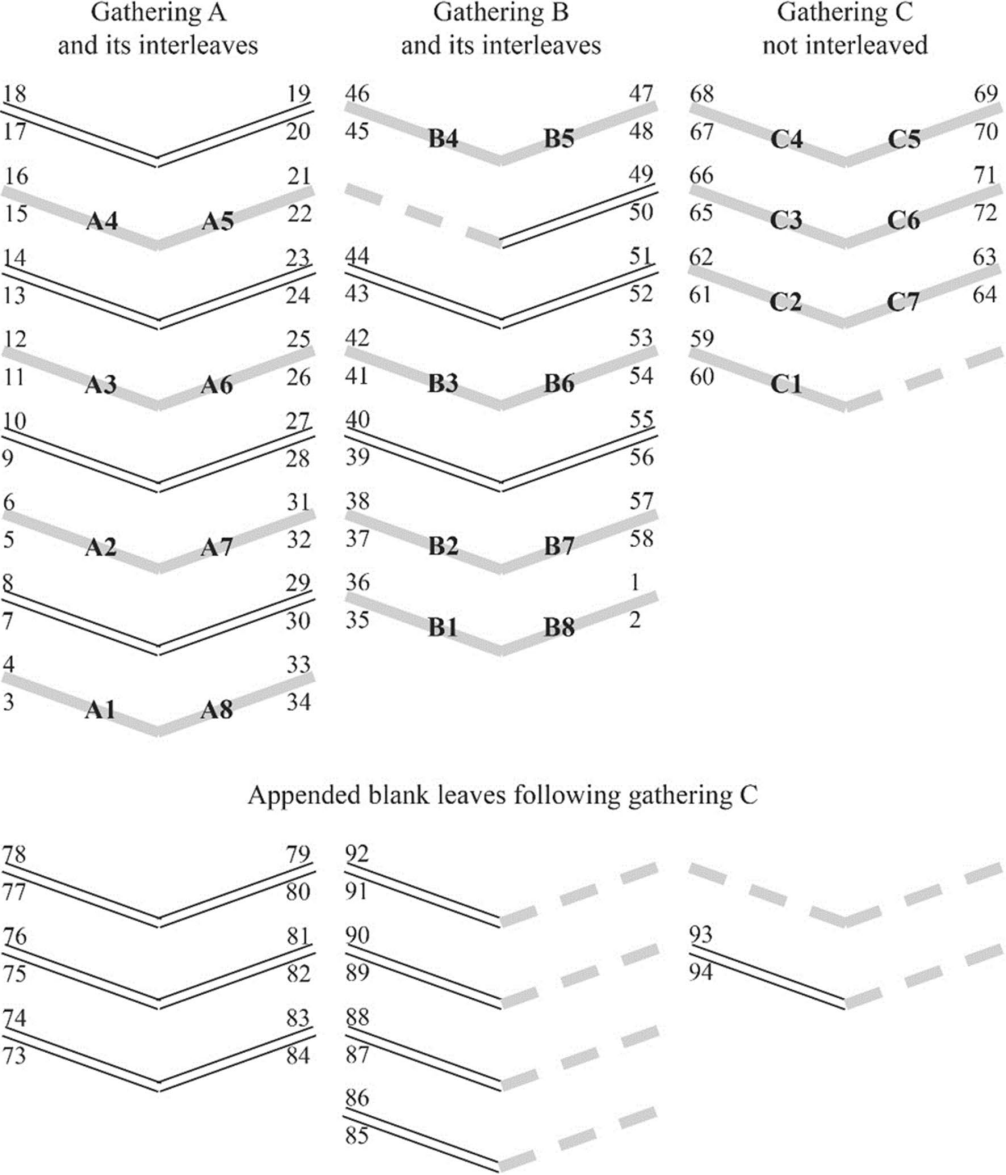
Structure of the Almanac gatherings, showing leaf pairs. The double lines represent blank leaves, solid lines represent printed leaves, and dashed lines represent leaves now missing

The printed elements of the Almanac consisted of 48 unnumbered pages in three octavo (8-leaf) gatherings (A, B and C). Additionally, 14 blank interleaves plus an unknown number of appended blank leaves provided space for the purchaser’s notes and memoranda; 11 of these appended leaves survive today. The structure of the Almanac is shown in Fig. 1. In this schematic depiction, the double lines represent blank leaves, solid lines represent printed leaves, and dashed lines represent leaves now missing. Leaves are identified by handwritten page numbers, and printed leaves are also identified by gathering and leaf number. The diagrams show gatherings as viewed from the book’s bottom edge with the book open. The base of the shallow “V” marks the fold or gutter through which the leaves would have been sewn when the volume was bound. In each gathering, the bottom leaf pair is outermost, the top pair innermost. So in the A gathering, p. 3 is the first, and the conjugate p. 34 is the last. Similarly, pp. 17–20 form the innermost pair of leaves or centerfold. Figure 1 shows that originally, each leaf in the volume was one half of a bifolium. However, some leaves became detached and are at present non-conjugate. Additional appended bifolia may have originally been present, and it is no longer possible to establish how many blank leaves were originally present at the end of the volume.

The book was sewn on three sewing supports. Five holes remaining in the spine folds (sewing stations) were recorded, evenly spaced and presenting darker-colored edges if compared to the color of the rest of the paper. It is likely that the three central ones were used for sewing the paper to the supports using linen thread and that the top and bottom holes were used for the kettle stitch. In addition, on the leaf now numbered 94–93, remnants of stitching and leather marks from a previous cover supported the conclusion that there had been an earlier leather binding, predating the nineteenth-century one.

The paper support used across the entire volume (the printed sheets, the interleaves, and the appended blank leaves) was hand-made from seventeenth-century rag paper, the only type of paper produced in the West at the time. Rag paper is primarily made of cotton, hemp, or linen fibers from recycled fabric and is recognizable for its pronounced texture along with the presence of laid lines and chain-lines left by the paper mold [18, 19].

Being octavo in format, the pages had vertical chain-lines and the watermarks were quartered across the upper gutter and the upper edge of four leaves, making it difficult to reconstruct the full watermark. However, one watermark was shared by both a printed page (pp. 47–48) and several blank leaves (interleaves 23–44, 51–52, and 55–56; appended blank leaves 77–78 and 85–86), suggesting that at least in these cases, the same paper stock was used for both components. The watermark was reconstructed as a fleur-de-lys above a post-horn bounded by a scroll, of a generic variety typified by Gravell § 791 [20].

**Fig. 2:**
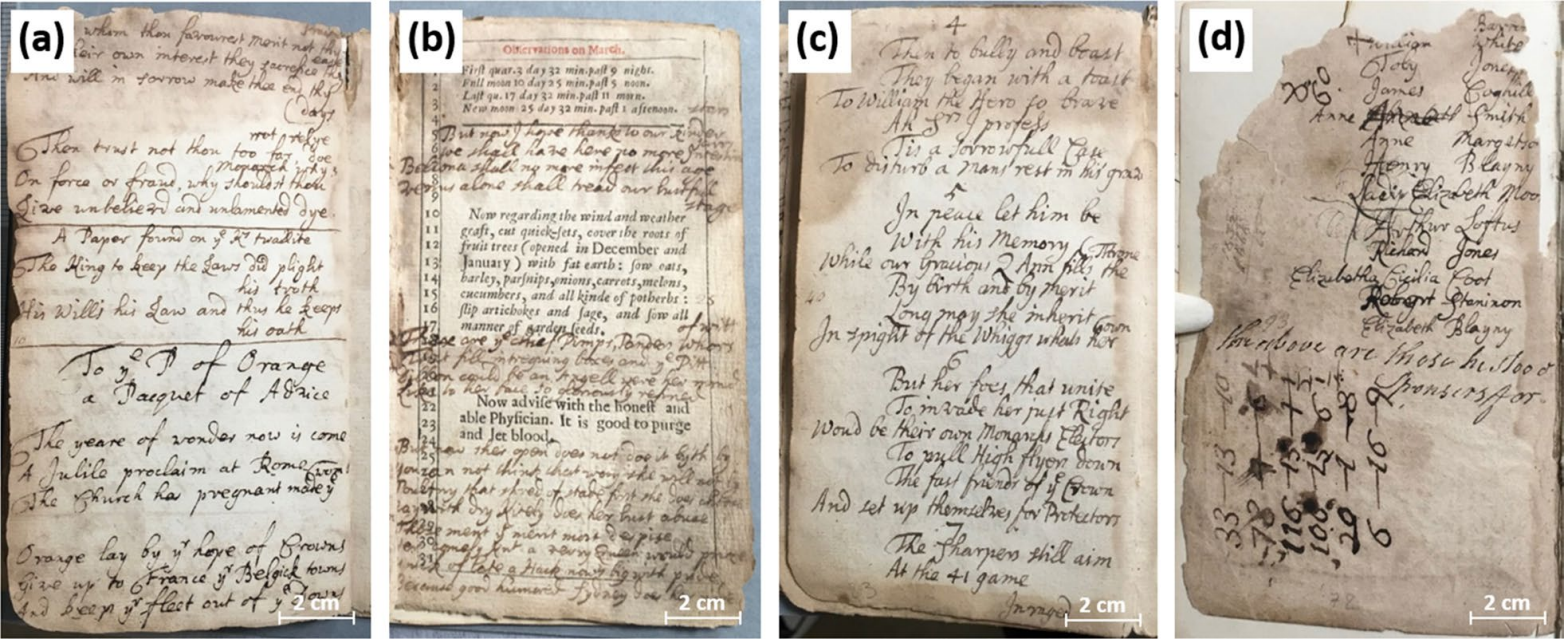
Pre-conservation photographs of damaged pages: **a** p. 10 (water stains), **b** p. 25 (mold), **c** p. 40 (tears), and **d** p. 93 (losses). Reproduction rights owned by the NLI. The same applies to all the following photographs

The volume’s leaves seemed to have been reordered on at least two subsequent occasions. On the first, the pages were hand-numbered, presumably following a reordering, which implies that some leaves had already become detached from the structure. During a further reordering, possibly after the acquisition of the volume in the early nineteenth century, some of the earlier reordering was revised. The current (nineteenth century) rebinding was seemingly assembled to protect the already damaged seventeenth century volume. The pages presented extensive damage with water stains, mold, tears, and losses as visible in Fig. 2a–d, respectively.

### The sequence in which the poems were inscribed: historical analysis

**Fig. 3:**
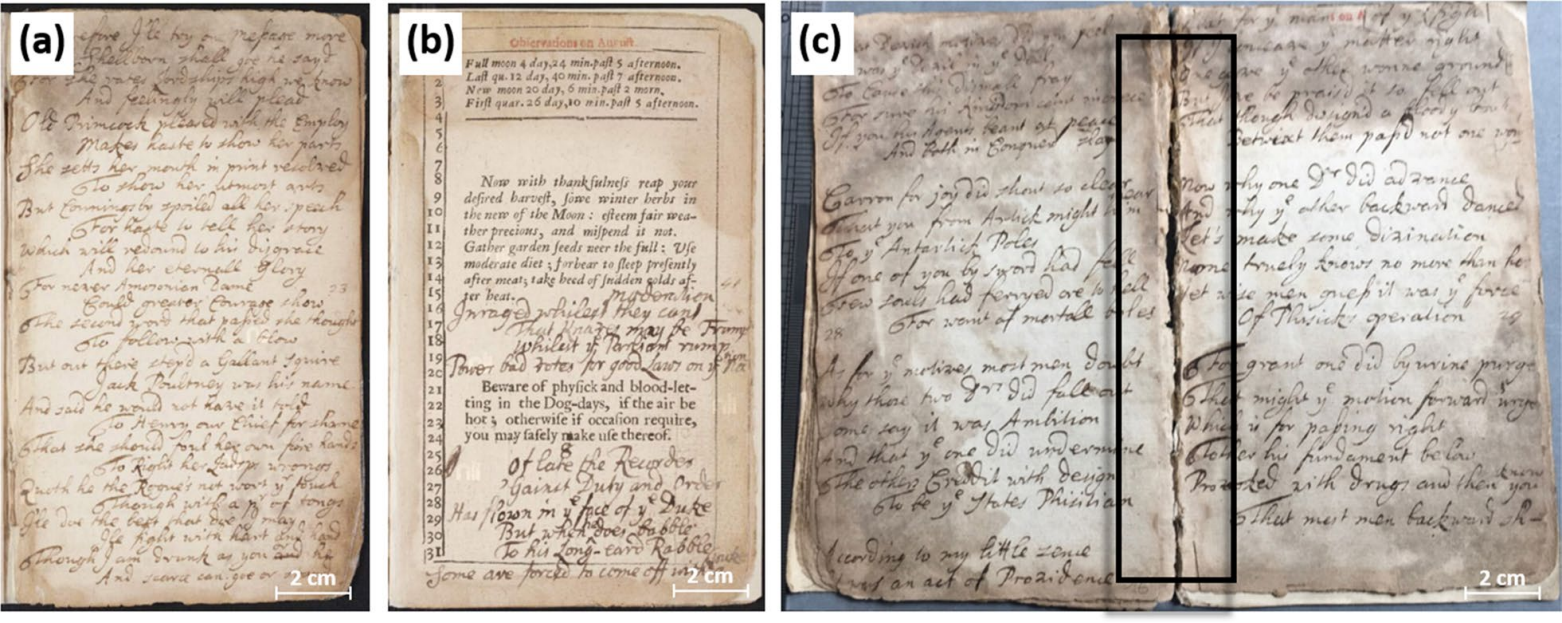
**a** The manuscript on an interleaf, p. 23; **b** the manuscript on a printed page, p. 25; **c** p. 28 and p. 29 before conservation. The black box shows the gutter with the writing running very close to the fold

In the late seventeenth and early eighteenth century, 19 poems were transcribed onto the blank interleaves and appended blank leaves as well as onto the blank areas of some of the printed leaves (see Fig. 3a, b). What remains a topic of debate is the exact point at which the leaves had become loose, allowing the scribe to write noticeably farther into the gutter than would normally be possible in a bound volume (Fig. 3c, and Additional file 1: Fig. S1).

Detailed observation of the pages allowed recording several characteristics about the prior reordering of the pages (for more details see Additional file 1). From a paleographical investigation of the handwritten sections, it appeared likely that a single scribe wrote all the poems. However, notable variations in handwriting size and spacing of the writing ink were observed across pages, leading to the assumption that the poems were entered in successive stints under varied circumstances, as the variations in ink elemental composition (discussed later) also suggest.

Based on the historical data regarding the poems’ composition and circulation dates (between 1674 and 1711), it appeared likely that the compiler gathered some of the poems towards the end of the seventeenth century, over a span of approximately 15 years, and added poems during the early years of the eighteenth century. Circulation dates, textual content, and codicological assessment suggested that the scribe began adding poems using several appended blank leaves that followed the C gathering of the printed Almanac (pp. 77–88). Once these blank leaves were filled with poems dated to the late seventies of the seventeenth century, the scribe continued writing on interleaves 7–10, 13–14, 17–20, and 23–24. The scribe then continued on the unprinted areas of the printed p. 25 (see Fig. 3b), before proceeding onto interleaves 27–30 (partly shown in Fig. 3c). These poems (pp. 7–30) are dated to the late eighties and nineties of the seventeenth century. The poems on pp. 89–92 date from 1699 to 1709, while a poem dated to 1711 is found on pp. 39–51.

What is puzzling is why the scribe, having reached the bottom of what is now p. 88, then continued that poem at the top of p. 7. A possible explanation is that the scribe only then realized that there was far more material to copy into the book than the remaining appended leaves could accommodate and simply chose that moment to go to the early interleaves of the volume and continue writing there. For the belated use of pp. 39–51, the readiest explanation is that no other such stretch of blank pages remained available as late as 1711.

Based on the above historical analysis, and especially on the poems’ composition date, an original sequence of poem insertion was proposed and it is shown in Table 1. This reconstructed sequence, as well as the open questions associated with the proposed order, established the basis for XRF and SOM analyses.

**Table 1:** Proposed original order in which the poems were transcribed in the Almanac

Page	Poem title	Composition date
77–78	Upon Nothing by y[e Earl o]f Ro[chester]	1678?
79–82	The Catholique Ballad or An Invitation to Popery	1674
83–87	Room for a Ballad, or A Ballad for Rome	1674
87–88	On Rome’s Pardons by the E. of R.	1675–80
88 and 7	On the Composeing of a Prayer for the Unborne Prince of Wales	1688
7–9	The Miracle	1687
9–10	A Paper Put in the K’s Shoo	1687?
10	A Paper Found on the K’s Twallite [toilette]	1685–88
10, 13	To the P of Orange: A Pacquet of Advice	1688
14, 17	The Pacquet Boat Returned	1688
17–19	The Gentlemen at Larges Litany	1692–93?
19–20, 23–24	To the Tune of Chivie Chace	1692–93
24–25, 27	Mrs Butler to Mrs Bracegirdle	1692–93?
27–30	The Duel between 2 Phisitians	1693
30	A Pascall [pasquil] Lately Come from France	1696
89–90	[The Picture of a Dublin Beau]	1699
90–91	A Fable, yet a True Story	1700-01
92	The Thanksgiving	1709?
39–41, 43–44, 49–51	The Whiggs Lamentation. A Soar of Their Own Scratching	1711

The question mark (?) indicates an uncertain composition date. For more details on the book structure see also Additional file 1: Table S1

### Elemental composition of inks and writing support: XRF analysis

A total of 104 XRF spectra, comprising 37 of blank areas and 67 of handwritten inks, were taken during this study. XRF measurements were performed in the NLI laboratories, during the conservation of the Almanac, when the book was in a disbound state and access to areas otherwise not accessible (i.e. the areas close to the book spine) was possible. XRF spectra were obtained for each handwritten page of the book. In cases where paleographical analysis suggested a change of poem or ink, multiple XRF spectra were captured per page. XRF analysis was also conducted on the blank areas of the paper to evaluate the elemental composition of the underlying support.

**Fig. 4:**
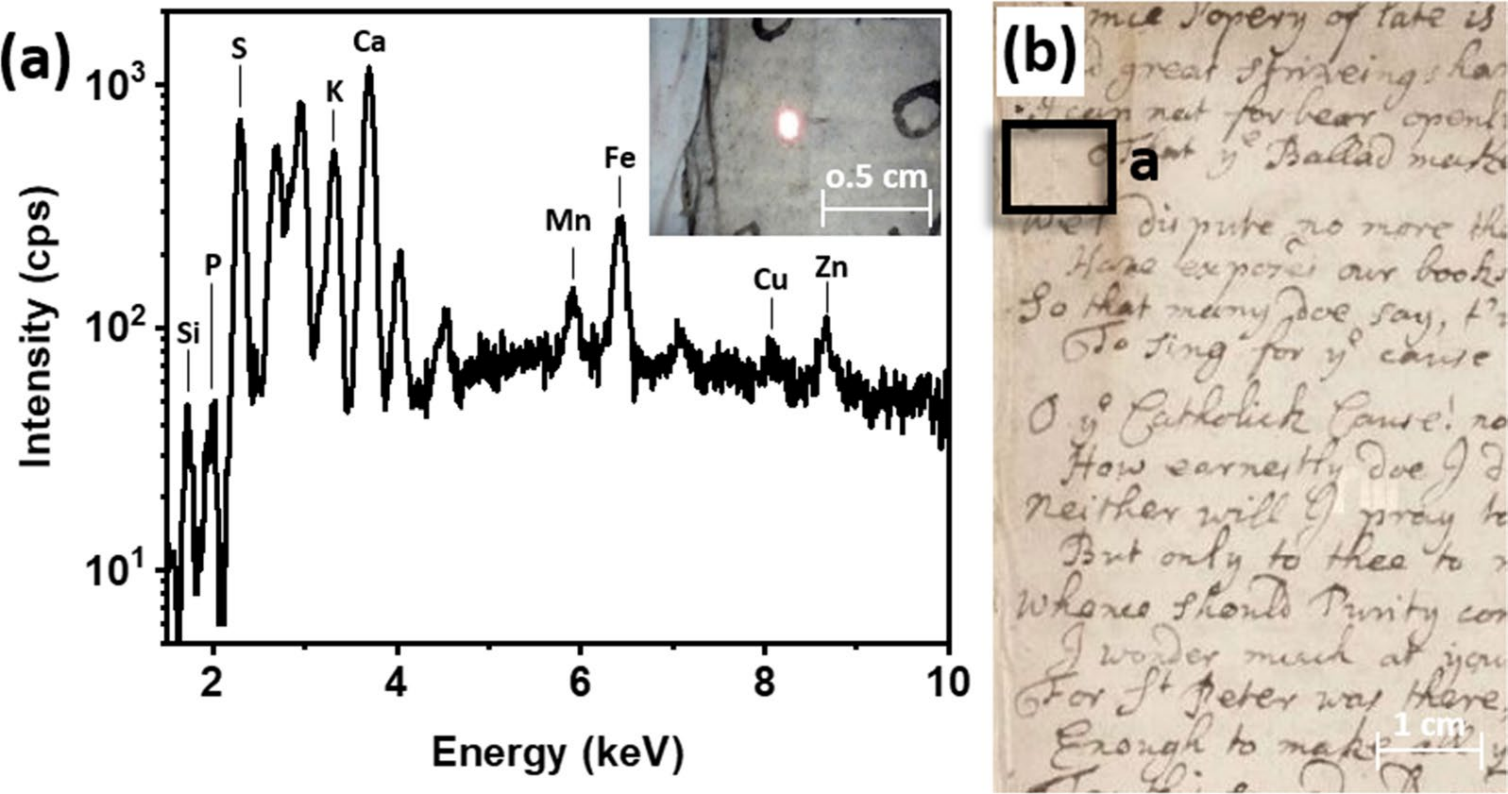
**a** XRF spectrum of blank paper taken at p. 79; **b** photograph (detail) of the area where the XRF spectrum was taken, highlighted by the red box

The XRF spectra of blank paper support recorded across the volume showed similar elemental features, with only small variations in elemental peak intensities despite the water damage observed. An example of an XRF spectrum of blank paper is shown in Fig. 4a. It was characterized by the presence of silica (Si), phosphorus (P), sulfur (S), potassium (K), calcium (Ca), manganese (Mn), iron (Fe), copper (Cu), and zinc (Zn).

The presence of Ca was attributed to calcium-based compounds known to be used for inducing fermentation of rags. These could include lime (calcium hydroxide, Ca(OH)_2_) or milk [21]. Considering the concomitant presence of S and Ca, gypsum (CaSO_4_ × H_2_O), a common ingredient used in paper making, could not be excluded. Ca could also be linked to the use of fillers such as calcium carbonate (CaCO_3_), commonly employed in paper production [21]. The presence of S and K, often detected at similar or higher intensities than Ca, suggested the possible use of alum (potassium/aluminum sulfate, KAl(SO_4_)_2_·12H_2_O) liquor in the sizing of the paper. Records from the early seventeenth century indicate the addition of alum to gelatin size, as a preservative and stabilizer of the viscosity of the sizing liquor [22]. Additionally, alum salts were used to reduce the absorbency of the paper, by depositing in the interstices of paper fibers, leading to better writing performance [21]. Interestingly, alum-treated papers are more susceptible to ageing, due to the increased acidity, which leads to the degradation of cellulose through acid-catalyzed hydrolysis [23]. Calcium-based compounds were used, among other reasons, to counteract and neutralize the acidity of alum-treated paper, thereby providing a protective effect [24]. Accordingly, the most degraded and brittle leaves of the Almanac showed higher intensities of K compared to calcium Ca, in agreement with the observations above. It is important to note that the fragility of the water-damaged leaves may be attributed to both the higher K concentration and to the water-induced dissolution of the gelatin size, which served as a protective layer from environmental agents. Therefore, the weakening of the paper could potentially be a result of these combined factors. Fe was observed in all recorded spectra and could be attributed to the presence of iron alum (FeAl(SO_4_)_2_ × 12H_2_O). Si was consistently detected though the book. The presence of Si in rag paper is reported in literature to be associated with the presence of straw, high in silicon dioxide (SiO_2_) [25]. Cu and Zn in minor concentrations were also identified in the XRF spectra. The presence of these elements could be related to the paper-production process, such as impurities from the water used for making the paper poultice or from contact with metallic tools—beater roll, mold, or press—employed during paper production [21]. Additionally, it is possible that S, K, Fe, Cu, and, Zn migrated from the printing and writing inks due to the extensive water damage observed.

**Fig. 5:**
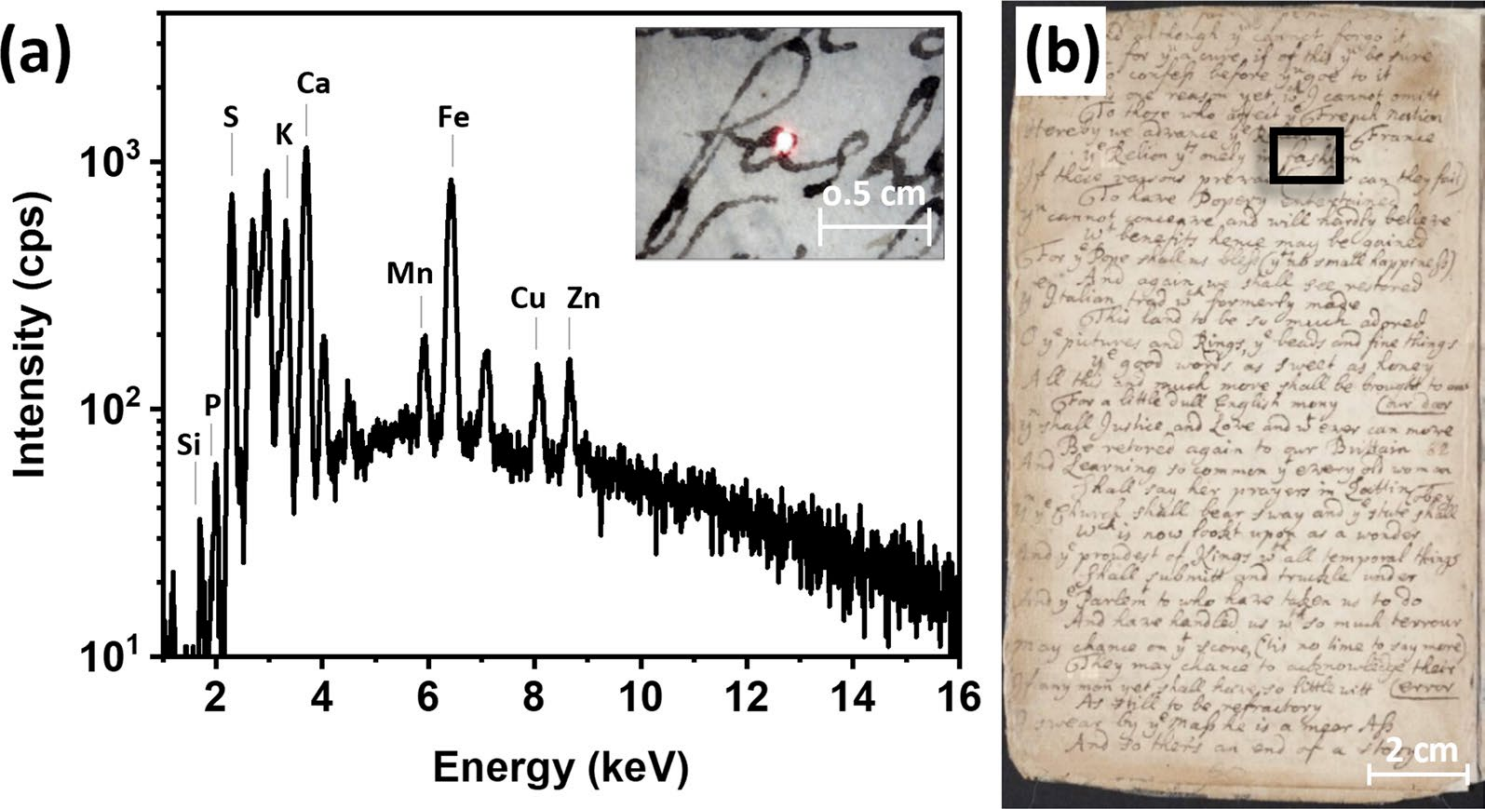
**a** XRF spectrum of an inked area, p. 82. Inset shows a microscopy picture of where the XRF spectrum was taken; **b** photograph of p. 82. Black box shows the area of analysis

In parallel with blank spots, spectra of handwritten text were taken. Inked areas that sufficiently encompassed the entire spot size (~ 1 mm) were chosen for analysis. A representative XRF spectrum of an ink used for the handwriting is shown in Fig. 5a.

The spectrum revealed the presence of several elements, including Si, P, K, Ca, Mn, Fe, Cu, and Zn. Si, P, and Mn were found at similar intensities in both the ink spectra and the paper spectra, suggesting that they were not diagnostic of the ink under analysis. On the other hand, S, K, Ca, Fe, Cu, and Zn exhibited higher peak intensities compared to the blank paper, possibly indicating their association with the ink applications. The combination of S, Fe, Cu, and Zn suggests the presence of vitriol, a mixture of hydrated metal (Fe, Cu, and Zn) sulfates, associated with iron-based inks, one of the standard ink formulation extensively used in Europe between the fifth and nineteenth century [26–28]. These inks were obtained by combining vitriol with polyphenolic compounds such as tannic acid or gallic acid. The reaction resulted in the formation of dark iron-polyphenol complexes, contributing to the distinctive dark color of iron-based ink [8]. The presence of K was attributed to the presence of gum arabic, commonly used in inks to improve viscosity and reduce bleeding [29]. It is worth noting that the presence of K may also be attributed to the presence of iron sulfate, as reported in studies on the chemical composition of iron-based inks [30, 31]. It was not possible to explain the increased Ca intensity in all the inked areas analysed. Further research and investigation is required to understand the reasons behind the increased Ca intensity in those particular areas.

### The sequence in which the poems were inscribed: XRF analysis of three case studies

Following the general classification of writing inks as iron-based inks, a comprehensive qualitative elemental analysis was conducted on the spectra. The primary objective of this analysis was to explore the various composition of the writing inks, with a particular focus on the vitriol constituent elements. This examination aimed to find out whether evidence supports the order of poem insertion suggested by the historical analysis. Notably, previous studies have highlighted the significance of observing variations in content of Mn, Cu, and Zn (relative to Fe) in XRF spectra of inks. These ratios are specific to each vitriol source or extraction method [29], which allows for the identification and classification of different inks [32–34]. Three case studies further illustrate these findings.

**Fig. 6:**
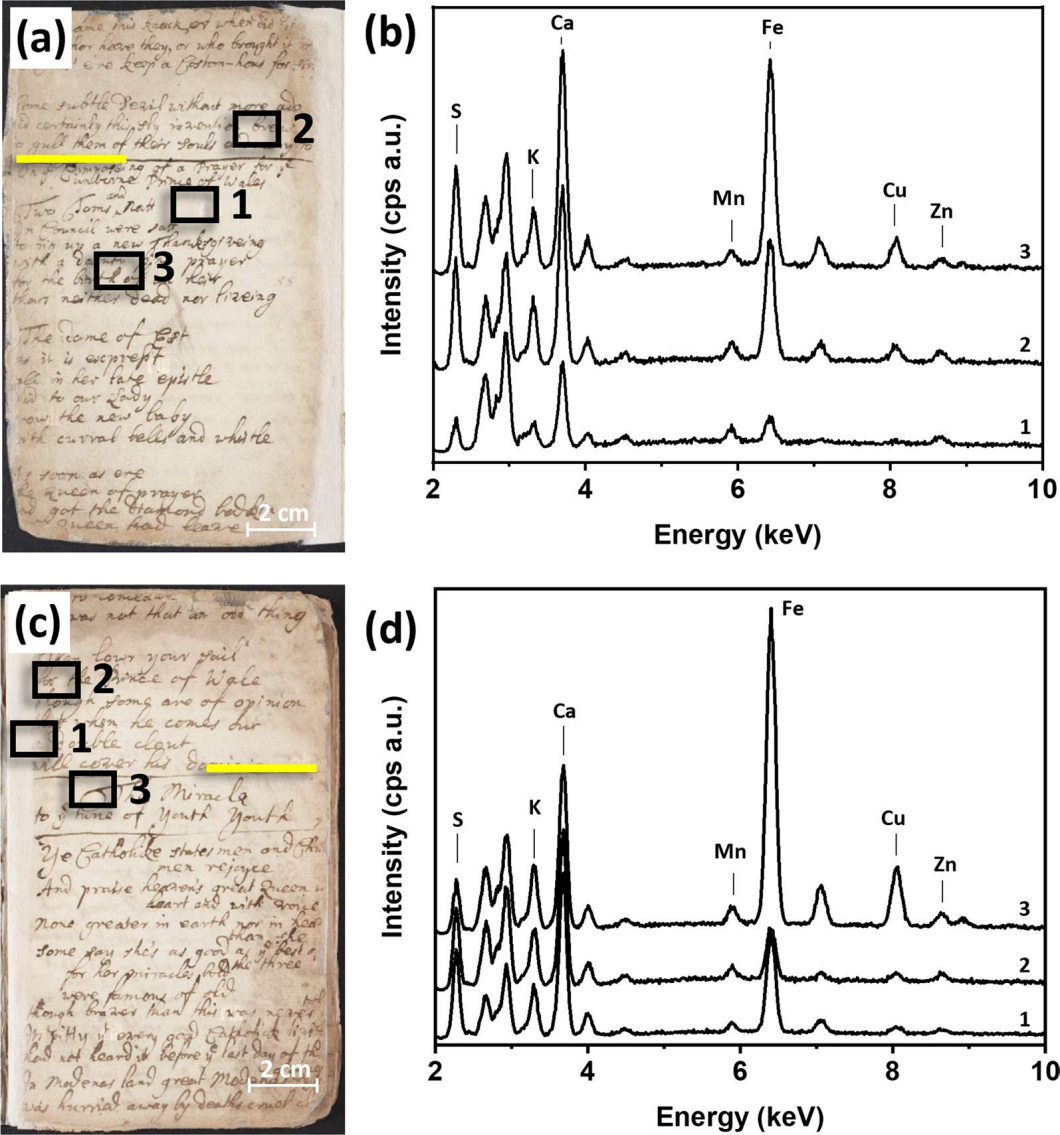
**a** Photograph of p. 88; **b** XRF spectra of paper and inks in poem “On Rome’s Pardons by the E. of R.” (1675–80) on p. 88. 1: blank paper; 2: ink in upper section; 3: ink in lower section; **c** photograph of p. 7; **d** XRF spectra of inks in poem “On the Composeing of a Prayer for the Unborne Prince of Wales” (1688), p. 7. 1: blank paper; 2: ink in upper section; 3: ink in lower section. In the photographs, the yellow line shows the break between the two different poems and the black boxes show where the XRF analysis was performed

The first case study focused on the proposed starting point of the transcribed text, where the scribe filled pp. 77–88 and continued writing on interleaf p. 7. On p. 88 (Fig. 6a), it was discernible that a horizontal line marked the conclusion of the poem “On Rome’s Pardons by the E. of R.,” which circulated between 1675 and 1680, followed by the commencement of “On the Composeing of a Prayer for the Unborne Prince of Wales” (1688). XRF spectra collected for these two segments exhibited similar compositions (Fig. 6b, spectra 2 and 3), particularly a constant low intensity for Zn, which was not considered indicative of the ink applications as it was detected as well in the blank paper, and a low peak intensities for Cu. This was observed also for all the previous pages (pp. 77–88) and suggested that these areas were likely penned using the same ink or inks of very similar composition. A similar trend was observed in the upper section of p. 7 (Fig. 6c). This consistency in elemental composition of ink between page 88 and 7 supported historical and codicological data.

When examining p. 7 (Fig. 6d), several differences between the text in the first and second half of the page became apparent. These differences included variations in thickness of the ink line and size of writing. By comparing the elemental composition of these two sections (Fig. 6d, spectra 2 and 3), the peak intensities of Cu was observed to be higher if compared with the previous cases, possibly suggesting that two different inks were used to write the first and second half of p. 7.

**Fig. 7:**
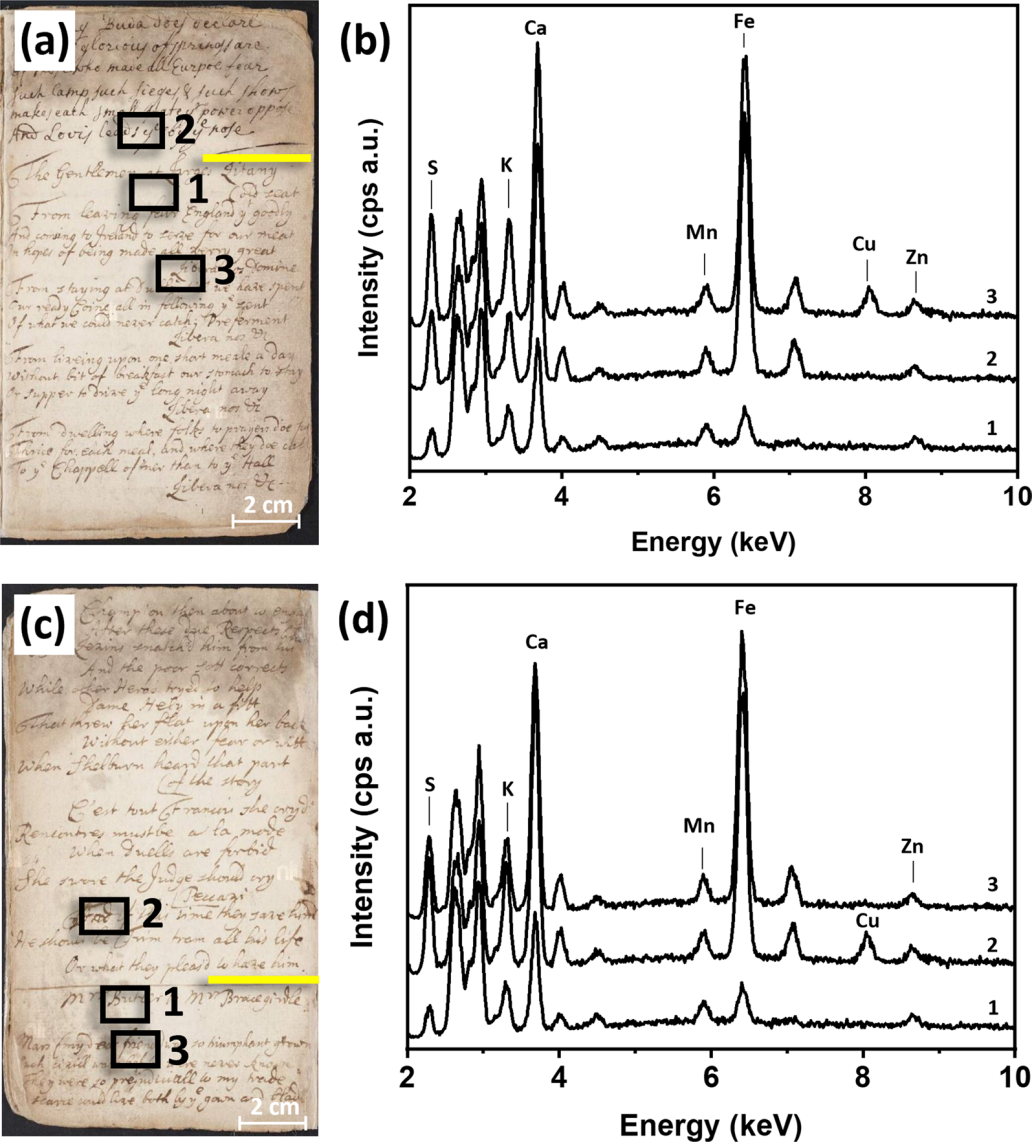
**a** Photograph of p. 17; **b** XRF spectra of paper and inks in poem “The Pacquet Boat Returned” (1688), p. 17. 1: blank paper; 2: ink in upper section; 3: ink in lower section; **c** photograph of p. 24; **e** XRF spectrum of ink in poem “To the Tune of Chivie Chace” (1692–93), p. 24; **f** XRF spectra of paper and inks in the poem “Mrs Butler to Mrs Bracegirdle” (1692–93), p. 24. 1: blank paper; 2: ink in upper section; 3: ink in lower section. In the photographs, the yellow line shows the break between the two different poems and the black boxes show where the XRF analysis was performed. The inset in the XRF spectra show a magnified image of the analyzed area

The second case study was related to the phase of inscription spanning pp. 7 to 31. The codicological assessment did not uncover discernible features that would indicate a disruption or break in the writing process. However, XRF analysis revealed a discontinuous elemental composition within the writing ink (see Fig. 7). All these leaves presented a constant low peak intensity for Zn, therefore that element was not considered indicative of the ink applications. Figure 7b exhibits XRF spectra taken from blank paper (spectrum 1), the ink in the upper section (spectrum 2), and ink in the lower section (spectrum 3) of p. 17. An horizontal line marks the conclusion of the poem “The Pacquet Boat Returned” (1688). The ink used for writing this poem contained ink diagnostic peaks corresponding to S, K, Fe, and Cu. In contrast, the XRF spectrum taken in the lower section of p. 17, below the horizontal line and featuring the poem titled “The Gentlemen at Larges Litany” (1692–93), was characterized by S, K, and Fe only, suggesting that a different ink was possibly used for penning this section. What is apparently the same ink continued to be used until the completion of the poem titled “To the Tune of Chivie Chace” (1692–93) (first half of p. 24, Fig. 7d, spectrum 2). In contrast, a new ink composition, featuring S, K, and Fe only, was observed in the second half of p. 24 in the “Mrs Butler to Mrs Bracegirdle” poem (from 1692 to 1693, Fig. 7d, spectrum 3).

**Fig. 8:**
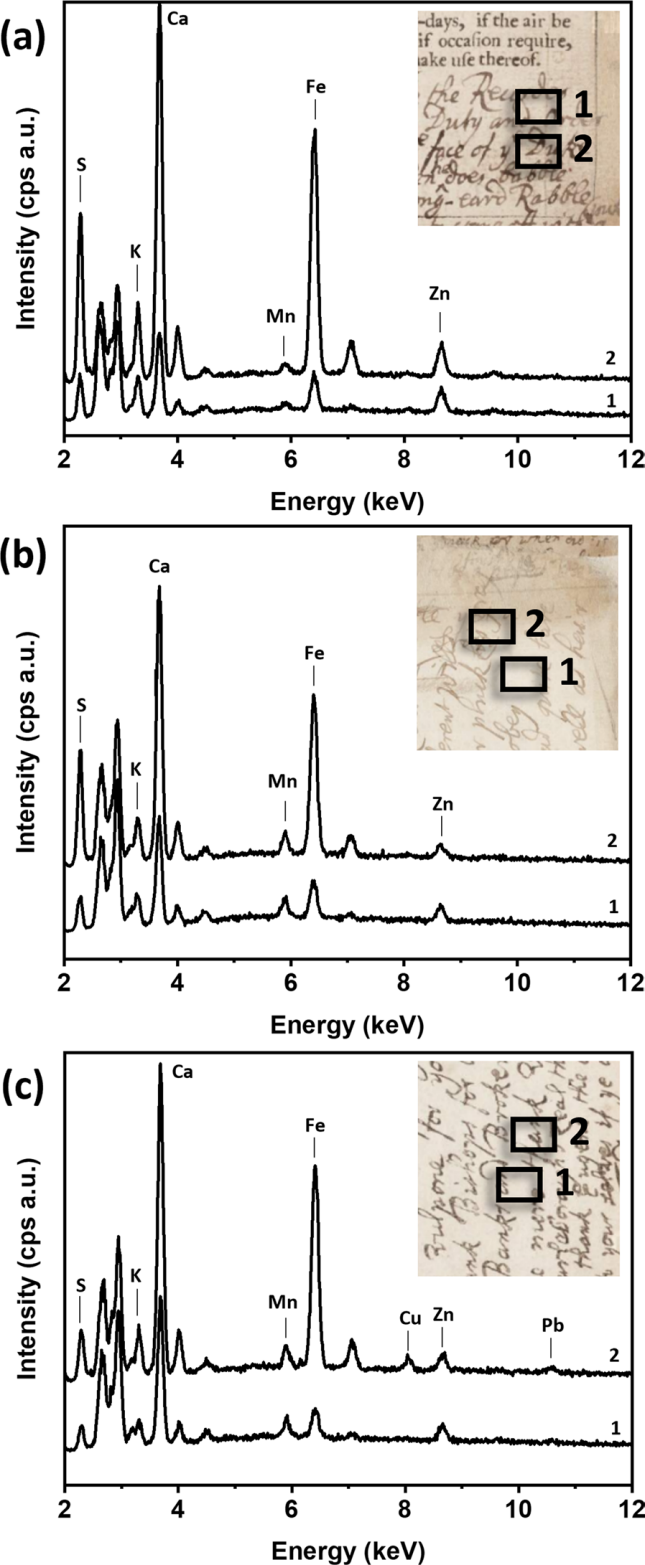
**a** XRF spectra of blank paper (1) and ink (2) in “The Whiggs Lamentation. A Soar of Their Own Scratching”, p.41; **b** XRF spectra of blank paper (1) and ink (2) in “The Picture of a Dublin Beau”, p 90; **c** XRF spectra of blank paper (1) and ink (2) in “The Thanksgiving.”, p. 92. The inset in the XRF spectra show a magnified image of the analyzed area and black boxes show the areas where XRF analysis was performed

The third case study focused on the analysis of the most recent additions to the Almanac, dating from the end of the 17th to the early years of the eighteenth century. Specifically, the following poems were analyzed: “The Whiggs Lamentation. A Soar of Their Own Scratching”, dated 1711, pp. 39–51 (p. 41 in Fig. 8a); “The Picture of a Dublin Beau”, from 1699, pp. 89–90 (p. 90 in Fig. 8b); “A Fable, yet a True Story,” from 1700 to 01, pp. 90–91; “The Thanksgiving,” probably from 1709, p. 92 (Fig. 8c).

In terms of ink composition, the XRF analysis of the ink on p. 41 (Fig. 8a, spectrum 2), which represents the inked areas of “The Whiggs Lamentation. A Soar of Their Own Scratching”, revealed the presence of only S and Fe. The high concentration of Zn was also detected in the XRF spectra of the unwritten paper, making it difficult to solely attribute it to the ink. Nonetheless, the consistency in ink composition throughout the poem on pp. 35–51 supports the findings of previous case studies, indicating that the writer likely used the same ink for each individual poem or small groups of these.

Pp. 89–90–91, compared with pp. 39–51, show variations in thickness of the ink line and size of writing. No noticeable differences were observed in their ink elemental fingerprint. Once again, the ink in that section (pp. 89–90–91) was characterized by the presence of S and Fe, with Zn being ascribable to the paper content (see representative XRF spectrum in Fig. 8b, spectrum 2). However, it is important to note that the elemental composition of the ink in p. 92 (Fig. 8c) has shown discontinuity from the previous short poems in pp. 89–90–91, displaying low peak intensities for Cu and lead (Pb). This suggests that a different ink may have been used for the poem “The Thanksgiving.”

In summary, the performed XRF analysis showed that different types of inks were used to compile the poems. Based on the available information, it is likely that all these short poems were written sequentially, roughly adhering to a chronological order. However, they were likely transcribed in several stints, a new stint sometimes entailing a different ink. This suggests a pattern of writing and compilation that deviates from a continuous, uninterrupted flow, and rather follows a periodic grouping approach with a variety of inks over time.

### XRF quantification and statistical analysis

The quantification of elements present in the XRF spectra of both blank paper and inked areas was obtained using the FP algorithm integrated in the PyMca software [16]. This approach facilitated the extraction of quantitative values for the identified elements (Si, P, Cl, S, K, Ca, Mn, Fe, Cu, and Zn) in each recorded spectrum. To account for the inherent variability resulting from the heterogeneity of ink applications and paper substrate, and to enable easier comparison between spectra, it was decided to express elemental concentrations relative to Fe.

Compared to other popular clustering techniques, such as K-means [35], SOM was selected for its ability to identify non-linear relationships between the samples, as it uses a neural network approach that can capture and represent complex data structures in a lower-dimensional space while preserving the main features of the original data. This capability was well-suited for the three case studies, as the variability of elemental peak intensities, relative to each other in various analysis spots, is influenced by multiple factors including composition, acquisition geometry, and thickness of the analysed ink.

In the context of XRF data analysis, SOM maps took the form of a 2D grid-like structure consisting of neurons. Each neuron represented a specific location in the map, and its weight vector corresponded to the elemental composition associated with that neuron. The statistical analysis pipeline began with feature extraction using Principal Component Analysis (PCA) on a dataset of 54 selected XRF spots, which identified Cu and Zn as the main chemical elements explaining the majority of the variance. This result was expected, as these two elements were already used in prior qualitative analysis as representatives of the vitriol sources. The elemental concentrations of Cu and Zn were then used to iteratively adjust the weights of the neurons during the training phase, which employed the Kohonen algorithm. This adjustment process involved computing the similarity of concentrations (W_Cu_/W_Fe_ and W_Zn_/W_Fe_) to the weight vectors associated with each neuron, with the goal of capturing and organizing the elemental composition patterns of the samples.

**Fig. 9:**
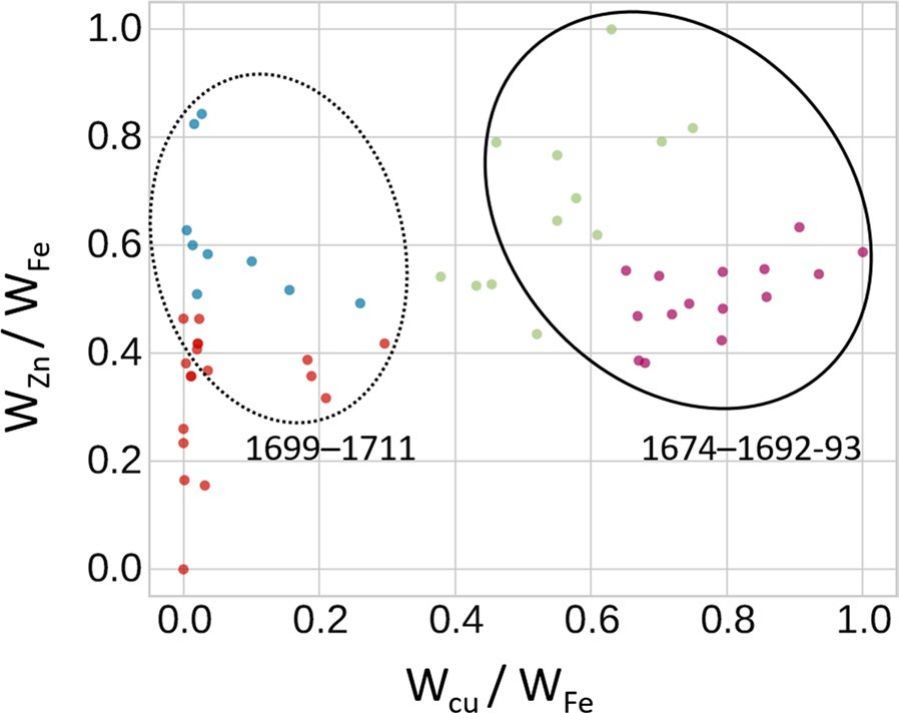
2 × 2 SOM-based clustering showing the date of composition of the poems

The clustering in Fig. 9 depicts four groupings, indicated by different colors, broadly representing four classes of inks. The green dots represent inks with a medium content of Cu and a high concentration of Zn (W_Zn_/W_Fe_ > W_Cu_/W_Fe_), while the purple dots represent inks with a high content of Cu and medium content of Zn (W_Cu_/W_Fe_ > W_Zn_/W_Fe_). The solid-line circle encompasses these two clusters as they make up the majority of the analyzed inks in the poems written during the early stage of the transcription. These satiric verses date from 1674 to 1692–93 (pp. 77–88, 7–17, 24–31, previously approached in case study 1). On the other hand, the dash-line circle groups the majority of analyzed areas from poems written from 1699 to 1711 (pp. 39–51 and pp. 89–92; case study 3). Within this area, the algorithm recognized two groups: one comprising inked areas with low concentration of both Cu and Zn (W_Cu_/W_Fe_ ≈ W_Zn_/W_Fe_, represented by red dots), and another group with low content of Cu and high concentration of Zn (W_Cu_/W_Fe_ < W_Zn_/W_Fe_, represented by blue dots). Interestingly, the spots representing the ink analyzed in case study 2 (pp. 17–24) were scattered outside the outlined circles. The findings from the analysis provided evidence supporting the reliability of SOM maps as a robust method for the initial categorization of data on elemental concentration of inks. It is important to note that when a larger amount of data is incorporated into the SOM maps, they are even more dependable for effectively organizing and categorizing complex datasets. While this approach was applied to Harward’s Almanac only, it is expected to be tested on larger datasets which comprise thousands of XRF spectra of ink.

## Conclusions

We have presented a transdisciplinary approach to the investigation of Harward’s Almanac, an Irish almanac of extreme rarity preserving important and, in some cases, unique texts of late seventeenth-century Irish poems. The investigation was conducted while the book was in a disbound state during conservation. This allowed historians to gain unique insight into the book’s manufacture, its subsequent binding and rebinding, and the reordering of the leaves as well as to formulate a hypothesis on the original order of the poems’ entry. The complementary XRF analysis performed on each handwritten page was key to confirming the validity of the historical hypotheses on the poems’ inscription and dating based on codicological and paleographic investigation. Finally, statistical analysis of all XRF data allowed the identification of four classes of iron-based inks, based on variable quantities of Cu and Zn. The statistical clustering allowed easy bi-dimensional visualization of all collected XRF data and provided further insights into ink similarities, which could be associated with date of circulation.

### Supplementary Information

**Additional file 1.** Semi-quantitative analysis of XRF data. Details of the reordering of the pages from codicological studies.

## Data Availability

The data that support the findings of this study are available from the corresponding author, DI, upon reasonable request. Python code for the statistical analysis of the elemental concentration values of inks is available at https://bit.ly/almanac-inkandskin.
