# Supplementary material for: Establishing the original order of the poems in Harward’s Almanac using paleography, codicology, X-ray fluorescence spectroscopy, and statistical analysis
Source: Herit Sci. 2023 Dec 15;11(1):265. doi: 10.1186/s40494-023-01107-y (PMC10724310; doi:10.1186/s40494-023-01107-y)
Supplement: Supplementary file 1 — Additional file 1. Semi-quantitative analysis of XRF data. Details of the reordering of the pages from codicological studies. [file 40494_2023_1107_MOESM1_ESM.docx]

**Additional file 1**

Establishing the original order of the poems in the Harward’s Almanac using paleography, codicology, X-ray fluorescence spectroscopy, and statistical analysis

Veronica Biolcati ^1,5^, James Woolley ^2^, Élodie Lévêque ^3^_,_ Andrea Rossi^4^, Anna Hoffmann ^5^, Andrea Visentin ^4^, Pádraig Ó Macháin ^5^, Daniela Iacopino *^,1^

^1^ Tyndall National Institute, University College Cork, Lee Maltings Complex, Dyke Parade, T12R5CP Cork, Ireland

^2^ Department of English, Lafayette College, Easton, PA 18042, United States

^3^ Centre de Recherche Histoire Culturelle et Sociale de l'Art (HiCSA), Université Paris 1 Panthéon-Sorbonne, 2 rue Vivienne, 74002 Paris, France

^4^ Centre for Research Training in Artificial Intelligence, Western Gateway Building, School of Computer Science & IT, University College Cork, Western Road, Cork, T12 XF62, Ireland

^5^ Modern Irish Department, University College Cork, College Road, T12K8AF Cork, Ireland

*corresponding author email: daniela.iacopino@tyndall.ie

**Semi-quantitative analysis of XRF data**

Initially, the noise fluctuation of raw experimental data was processed with a Savitsky-Golay smoothing. This smoothing operation modelled the region of interest of the spectra (first channel 155, last channel 1000) using a polynomial. Also, to achieve an accurate estimation of the elemental concentration in XRF spectra, it is pivotal to effectively model and subtract the continuum background. Therefore, the net peak area of the elements was obtained after being corrected of the background. For this operation a SNIP (statistical nonlinear iterative peak clipping) of width 26 was used. Then, the peak shapes were examined in order to test if the fitting function was correctly modelling the raw spectra and was therefore suitable for the next step: the batch fitting. This fitting procedure, to minimize the difference between the fitting line representing the experimental data and the raw spectra, was reiterated until the best set of parameters was determined. Then the elements of interest (Si, P, Cl, K, Ca, Mn, Fe, Cu, and Zn) were selected. The X-ray tube emission profile for the ELIO spectrometer, and other parameters such as primary beam filter, the thickness of the beryllium window, and the active area of the detector, were provided to the authors via configuration files and accounted for. Experimental parameter such as time exposure, geometry of acquisition and the tube flux were in accordance with the setup used during data acquisition. After having obtained a good agreement between fitting line and experimental data, spectra were batch fitted using the PyMCA batch fitting tool. Batch fitting involves the automated integration of the area under each fluorescence peak, allowing for the evaluation of the individual element contributions to the spectrum acquired. Finally, to semi-quantify the elements of interest the RGB correlator tool was used. This tool allowed to calculate each element ratio normalized to Fe.

**Details of the reordering of the pages from codicological studies**

Detailed observation of the pages enabled the recording of several characteristics about the prior reordering of the pages. An interleaf, formerly conjugate with pp. 43-44, is missing (apparently torn out), though its spine fold, along with some handwriting, was visible when the volume was disbound for conservation. The poem on pp. 39-51 (with catchword on p. 44 leading to p. 49) is complete. When the pages were hand-numbered, the part-title to the second printed section, entitled “A Prognostication for the Year of Our Lord God 1666,” was mistakenly numbered 1-2 and evidently placed ahead of the actual title page, which is numbered 3-4. Leaf C1, evidently loose, was turned the wrong way and numbered 60-59. Leaf C7 was mislocated and numbered 63-64. These errors were corrected in the 19th-century rebinding. By that time, or perhaps later, leaf C8 was lost. The catchword “*From*” leading to C8 remains at the foot of leaf C7v. This lost leaf, which almost certainly ended the Almanac, was the conclusion of the section entitled “The High-Wayes of Ireland.” The interleaf bifolium consisting of pp. 7-8 and 29-30 once stood between pp. 4-5 and 32-33 (as shown in Figure S3), that is, not in its 19th-century position between *A*2-3 and *A*6-7 but between *A*1-2 and *A*7-8. Evidence of this is of two kinds. First, the lines of “The Duel between 2 Phisitians” are in the wrong order when the 29-30 interleaf is misplaced in its 19th-century position but in the correct order if it is moved to follow p. 32, as is readily seen from comparison of the Almanac text with that published in *The Counter-Scuffle* (Dublin: E. Waters for M. Gunne, 1708), sig. B7r-v (copy in Boston College library). Second, when the interleaf is moved to the correct position, ink from the interleaf is mirrored in offset on facing pages of the printed Almanac. For example, Figure S1a,b shows photos of pp. 6 and 9 side by side. It can be noticed that several ink spots in the inner margin of p. 6 are offset from areas of heavy ink on the facing manuscript interleaf, p. 9 on the printed page (see detail in Figure S1c). In order for the ink spots on p. 6 to mirror the accumulated ink on p. 9, there would have to have been some give in the binding, or the page may have been loose, allowing the interleaf to slip up and down relative to the printed leaf. Such looseness may explain why the edges of leaves in the book are often tattered. The structure was simply not securely held together with thread, even as early as the inscription of p. 9.


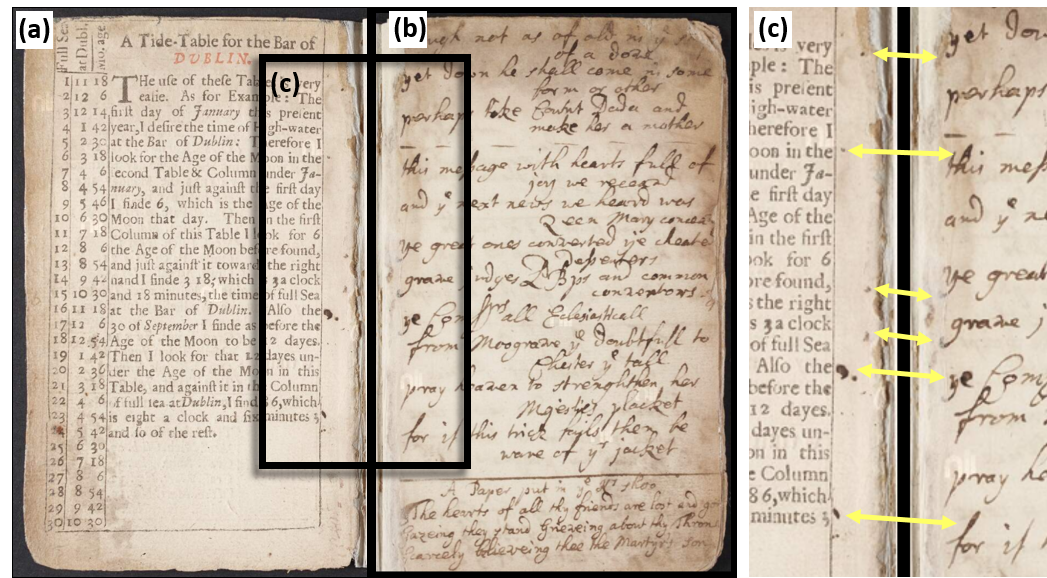


**Figure S1. (**a-b) Pre-conservation photographs of pp. 6 and 9; white box represents the location of the detail in (c); (c) Detail of the inner margins of pp. 6 and 9, side by side, showing the slight offset of the ink stains from the manuscript (p. 9, right) onto the printed page (p. 6, left). Yellow arrows indicates areas of ink offset.

Placing photos of pp. 32 and 29 side by side appears to reveal several small instances of ink offset from 29 onto 32 (see Figure S2). In the gutter of 32 opposite the lines for 21 and 26 May, there are spots of brown ink that appear to match the excess ink on the capital F of “For,” 6 lines from the bottom of p. 29, and the excess ink of the capital T of “Tother,” 3 lines from the bottom of 29. A small spot of brown ink in the outer margin of 32 against 30 May probably is a vestige of the completion of the last word on 29, the last letter or letters of which are now lost (“sh—t” or “sh—te”). In order for the ink spot between lines 6 and 7 of p. 32 to mirror the accumulated ink on the “d” of “passd” on p. 29, there would have to have been some play in the binding, allowing the interleaf to slip up and down relative to the printed leaf. In the 19th-century rebinding, the last leaf (pp. 94, 93) was turned over, reversing the original numberer’s sense of which side came first. An undetermined number of the final blank leaves following the last printed leaf C8 have disappeared, along with the missing C8.


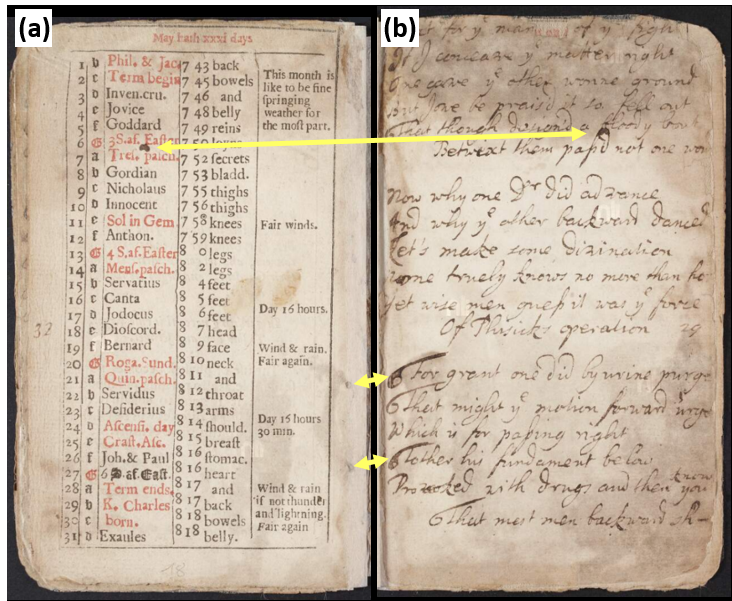


**Figure S2.** (a) Photograph of p. 32; (b) photograph of p. 29. Yellow arrows indicates areas of ink offset.

**Structure of the book, with leaves in their pre-19th-century order**

**Table S1.** The sometimes incorrect hand-numbering of the pages perhaps occurred in the late 18th or early 19th century. That mistaken ordering of the pages was the understandable consequence of wear and tear, loose and failing stitching, and the detaching and faulty relocation of several leaves or bifolia. Some of the mistakes were corrected for the 19th-century rebinding. In the table below, two additional ordering revisions are noted on the basis of the present investigation:

- the pages erroneously numbered 7-8 are correctly placed between pp. 4 and 5; and
- the pages erroneously numbered 29-30 are correctly placed between pp. 32 and 33.

The printed elements of the Almanac form three gatherings: the first (notionally *A*) is unsigned, the second signed “B,” and the third “C.” Page numbers use the handwritten pagination. Cells containing handwriting are highlighted in light gray.

| **Leaf** | **Page** | **Topic or title (portions on blank leaves are in *italics*)** |
| --- | --- | --- |
| *A*1 | 3  4 | [Title page]  Woodcut relating parts of the body to signs of the Zodiac |
|  | 7  7-8 | *On Rome’s Pardons by the E. of R.* (1675-80) [continued from p.88]  *The Miracle* (1687) |
| *A*2 | 5  6 | A Note to Know When the Four [law] Terms Begin and End  Tide-Table for the Bar of Dublin |
|  | 9  9-10  10  10 | *The Miracle* [continued from p. 8]  *A Paper Put in the K’s Shoo* (1687?)  *A Paper Found on the K’s Twallite* [*toilette*] (1685-88)  *To the P of Orange: A Pacquet of Advice* (1688) |
| *A*3 | 11  12 | A Table Whereby to Know the Age of the Moon  January hath xxxi days |
|  | 13  14 | *To the P of Orange: A Pacquet of Advice* [continued from p. 10]  *The Pacquet Boat Returned* (1688) |
| *A*4 | 15  16 | Observations on January  February hath xxvii days |
|  | 17  17-19 | *The Pacquet Boat Returned* (1688) [continued from p. 14]  *The Gentlemen at Larges Litany* (1692-93?) |
|  | 19-20 | *To the Tune of Chivie Chace* (1692-93) |
| *A*5 | 21  22 | Observations on February  March hath xxxi days |
|  | 23-24  24-25 | *To the Tune of Chivie Chace* [continued from p. 20]  *Mrs Butler to Mrs Bracegirdle* (1692-93?) |
| *A*6 | 25  26 | Observations on March  April hath xxx days |
|  | 27  27-28 | *Mrs Butler to Mrs Bracegirdle* [continued from p. 25]  *The Duel between 2 Phisitians* (1693) |
| *A*7 | 31  32 | Observations on April  May hath xxxi days |
|  | 29-30  30 | *The Duel between 2 Phisitians* [continued from p. 28]  *A Pascall* [*pasquil*] *Lately Come from France* (1696) |
| *A*8 | 33  34 | Observations on May  June hath xxx days |
| B1 | 35  36 | Observations on June  July hath xxxi days |
| B2 | 37  38 | Observations on July  August hath xxxi days |
|  | 39-40 | *The Whiggs Lamentation. A Soar of Their Own Scratching* (1711) |
| B3 | 41  41  42 | *The Whiggs Lamentation* [continued from p. 40]  Observations on August  September hath xxx days |
|  | 43-44 | *The Whiggs Lamentation* [continued from p. 41] |
|  | -- | [*interleaf deleted before pages were numbered*] |
| B4 | 45  46 | Observations on September  October hath xxxi days |
| B5 | 47  48 | Observations on October  November hath xxx days |
|  | 49-51  52 | *The Whiggs Lamentation* [continued from p. 44]  [*blank*] |
| B6 | 53  54 | Observations on November  December hath xxxi days |
|  | 55-56 | [*blank interleaf*] |
| B7 | 57  58 | Observations on December  Upon the Year, 1666 [poem] |
| B8 | 1  2 | Part title to “A Prognostication for the Year of Our Lord God 1666”  Of the Four Quarters of the Year (catchword to C1) |
| C1 | “60” [59]  “59” [60] | Of the Eclipses this presen[t] year 1666 (catchword to C1v)  The Principal Fairs of Ireland |
| C2 | 61-62 | The Principal Fairs of Ireland [continued] |
| C3 | 65-66 | The Principal Fairs of Ireland [continued] |
| C4 | 67-68 | The Principal Fairs of Ireland [continued] |
| C5 | 69-70 | Fairs That Are upon Moveable Feasts |
| C6 | 71-72 | The High-Wayes of Ireland |
| C7 | 63-64 | The High-Wayes of Ireland [continued]; formerly placed after C2. C7v catchword (“*From*”) to C8, which is missing |
| C8 |  | [missing; presumably C8 was the final printed leaf of the Almanac] |
|  | 73-74 | [*financial jottings*] |
|  | 75-76 | [*financial jottings* + *blank*] |
|  | 77-78 | *Upon Nothing by y*[*^e^ Earl o*]*f Ro*[*chester*] (1678) |
|  | 79-82 | *The Catholique Ballad or An Invitation to Popery* (1674) |
|  | 83-87 | *Room for a Ballad, or A Ballad for Rome* (1675-80) |
|  | 87-88  88 | *On Rome’s Pardons by the E. of R.* (1675-80)  *On y^e^ Composeing of a Prayer for y^e^ Unborne Prince of Wales* [continuing to p.7] |
|  | 89-90  90-91 | [*The Picture of a Dublin Beau*] (1699)*,* title and first two lines lost  *A Fable, yet a True Story* (1700-01) |
|  | 92 | *The Thanksgiving* (1709?) |
|  | -- | [*leaf or leaves missing* *before pages were numbered*] |
|  | “94” [93]  “93” [94] | [*financial jottings*]  *List of “those he* *stood sponser for” (“he” presumably being the compiler)* |
